# Molecular Alterations and Putative Therapeutic Targeting of Planar Cell Polarity Proteins in Breast Cancer

**DOI:** 10.3390/jcm12020411

**Published:** 2023-01-04

**Authors:** Ioannis A. Voutsadakis

**Affiliations:** 1 Algoma District Cancer Program, Sault Area Hospital, Sault Ste. Marie, ON P6B 0A8, Canada; ivoutsadakis@yahoo.com or ivoutsadakis@nosm.ca; 2 Section of Internal Medicine, Division of Clinical Sciences, Northern Ontario School of Medicine, Sudbury, ON P3E 2C6, Canada

**Keywords:** cell polarity, metastasis, breast cancer, tetraspanins, scribble

## Abstract

Background: Treatment and outcomes of breast cancer, one of the most prevalent female cancers, have improved in recent decades. However, metastatic breast cancer remains incurable in most cases, and new therapies are needed to ameliorate prognosis. Planar cell polarity (PCP) is a characteristic of epithelial cells that form layers and is integral to the communication of these cells with neighboring cells. Dysfunction of PCP is observed in cancers and may confer a targetable vulnerability. Methods: The breast cancer cohorts from The Cancer Genome Atlas (TCGA) and the METABRIC study were interrogated for molecular alterations in genes of the PCP pathway. The groups with the most prevalent alterations were characterized, and survival was compared with counterparts not possessing PCP alterations. Breast cancer cell lines with PCP alterations from the Cancer Cell Line Encyclopedia (CCLE) were interrogated for sensitivity to drugs affecting PCP. Results: Among genes of the PCP pathway, *VANGL2*, *NOS1AP* and *SCRIB* display amplifications in a sizable minority of breast cancers. Concomitant up-regulation at the mRNA level can be observed mostly in basal cancers, but it does not correlate well with the amplification status of the genes, as it can also be observed in non-amplified cases. In an exploration of cell line models, two of the four breast cancer cell line models with amplifications in *VANGL2*, *NOS1AP* and *SCRIB* display sensitivity to drugs inhibiting acyl-transferase porcupine interfering with the WNT pathway. This sensitivity suggests a possible therapeutic role of these inhibitors in cancers bearing the amplifications. Conclusion: Molecular alterations in PCP genes can be observed in breast cancers with a predilection for the basal sub-type. An imperfect correlation of copy number alterations with mRNA expression suggests that post-translational modifications are important in PCP regulation. Inhibitors of acyl-transferase porcupine may be rational candidates for combination therapy development in PCP-altered breast cancers.

## 1. Introduction

Breast cancer is one of the most prevalent female cancers and affects more than 10% of women in their lifetime in western countries and about 300,000 women annually in the United States [1]. The average age at diagnosis is 64 years old, and 42% of cases are women younger than 60 years old [2]. Outcomes of the disease have improved in recent decades as a consequence of earlier diagnosis and improved treatments, and most patients diagnosed in a localized stage can expect to be cured. In contrast, patients who are diagnosed with metastatic breast cancer or with recurrence of metastases almost invariably succumb to their disease, despite the availability of newer targeted treatments derived from a better understanding of the molecular biology of the disease [3,4,5]. Breast cancer is a heterogeneous disease, and sub-type classification is an integral part of treatment in the clinic. Predictive biomarkers, such as ER and HER2, help to categorize breast cancers as ER positive, HER2 positive, or triple negative (not expressing either of the two receptors or the progesterone receptor) and also serve as predictive biomarkers for the respective targeted therapies. Therapies are tailored according to the molecular sub-type, and improvement of survival outcomes, especially in ER-positive and HER2-dependent breast cancers, has been obtained by targeting key molecular players in each sub-type [6,7,8,9]. More recently, targeted therapies have also become available for subsets of triple-negative breast cancers in the form of immunotherapies with checkpoint inhibitors or of PARP inhibitor treatments [10,11,12]. Despite this progress, about 43,000 women were projected to die from breast cancer in the United States in 2022 [1]. Hence, an unmet need for innovative therapies to improve outcomes in these patients exists.

Planar cell polarity (PCP) is one of the cell polarity pathways with ties in development and cancer. It interconnects with the Wnt pathway and is thus considered a branch of Wnt signaling independent of β-catenin [13]. PCP signaling contributes to securing the correct orientation of epithelial cells along the planar axis through the positioning of tetraspanins VANGL1 and VANGL2 (Van Gogh-like 1 and 2) and interaction with Wnt signaling. PCP and its component proteins, such as VANGL homologues and SCRIBBLE, encoded by gene *SCRIB*, are instrumental in the development of organs with highly specialized functions, such as the nervous system and the inner ear [14]. In mammary epithelia, PCP machinery is involved in alveologenesis induced in pregnancy and the ensuing lactation [15]. In migrating cancer cells, VANGL1 and VANGL2 occupy cell membrane domains opposite to Wnt/Fzd complexes, promoting protrusion formation for mesenchymal-like cell motility [16].

In breast cancer, VANGL1 co-localizes with SCRIBBLE and NOS1AP (Nitric Oxide Synthase 1 Adaptor Protein) in cellular protrusions and regulates the migration of cells [17]. The knockdown of SCRIBBLE and NOS1AP with shRNA reduces the migration of breast cancer cells in vitro. Thus, the VANGL/SCRIBBLE/NOS1AP PCP complex may be involved in breast cancer metastasis. The current study examines the role of core components of PCP in breast cancer and focuses on members of the VANGL/SCRIBBLE/NOS1AP complex, which display copy number alterations in a sizable minority of mammary carcinomas.

## 2. Methods

The breast cancer cohort of The Cancer Genome Atlas (TCGA) and the cohort of the METABRIC study were used in the analysis of breast cancer samples [18,19]. Molecular aberrations in the genes of interest were surveyed using the online platform cBioPortal for Cancer Genomics Portal (cBioportal; http://www.cbioportal.org, accessed 19 September 2022), a genomics site initially developed by Memorial Sloan Kettering Cancer Center (MSKCC) and currently maintained by MSKCC in collaboration with others [20,21]. cBioPortal is an open-access, user-friendly source that allows investigators to interrogate various genomic studies for any gene of interest. cBioportal includes data on mutations, copy number alterations (CNAs) and mRNA expression, as well as correlative clinical data [21]. TCGA uses the GISTIC (Genomic Identification of Significant Targets in Cancer) algorithm for the analysis of CNAs. In this algorithm, a gene is considered as putatively amplified if it obtains a score of 2 or above, while scores of −2 and below denote putative deep deletion [22]. mRNA expression measurements in TCGA were performed through three platforms (Agilent, Santa Clara, CA, USA, Affymetrix HuEx and Affymetrix U133A) [18]. For mRNA analysis, TCGA used the RSEM algorithm for the normalization of mRNA expression [23]. RSEM quantifies different gene abundance from single-end or paired-end RNA-Seq data without requirement for a reference genome. The expectation maximization algorithm used in RSEM is able to handle and correctly classify transcripts from different isoforms of the same gene, providing improved accuracy [23]. Results are expressed as the Log RNA sequences in Reads per Kilobase Million (RPKM).

Putative pathogenic implications of alterations in cancer genes of interest were derived from the OncoKB knowledgebase (www.oncokb.org, accessed 11 July 2022), which classifies listed genes as cancer-related genes, oncogenes or tumor suppressors [24].

The expression of PCP proteins of interest was evaluated in the Human Protein Atlas (proteinatlas.org, accessed 29 June 2022), a publicly available database created and maintained by Stockholm University and the Karolinska Institute [25]. The Human Protein Atlas contains microarray immunohistochemistry data for proteins in normal human tissues and in cancers of various origins and provides a semi-quantitative estimation of expressions.

The breast cancer cell lines collection of the Cancer Cell Line Encyclopedia (CCLE) was assessed through cBioportal (accessed 16 September 2022) [26]. Cell lines with alterations in PCP genes were compared with breast cancer cell lines without such alterations for recurrent features and concomitant molecular alterations.

Genomics of Drug Sensitivity in Cancer (GDSC; www.cancerrxgene.org, accessed 6 October 2022) is an open-access database maintained by Sanger Institute and Broad Institute [27]. GDSC contains information of array experiments testing drug sensitivities of cancer cell lines [27]. Two datasets, GDSC1 and GDSC2, are available, the former with experiments performed between 2009 and 2015 and using media alone in the negative controls (cell lines not exposed to drugs) and the latter with experiments performed after 2015 and using media with a vehicle (DMSO) in the negative controls. The database was queried for drug sensitivity of breast cancer cell lines with and without PCP alterations. In addition, dependencies of breast cancer cell lines on specific genes was obtained from the Depmap project, which contains data from CRISPR array screens and RNA interference array screens performed on cell lines from the Cancer Cell Line Encyclopedia (CCLE) [28,29,30,31].

Statistical comparisons of categorical and continuous data were carried out with Fisher’s exact test or the χ^2^ test and the *t*-test. The prognosis of the groups of breast cancer patients with or without PCP gene amplifications and mRNA over-expressions was evaluated by constructing Kaplan–Meier curves. The Log Rank test was used to compare Kaplan–Meier survival curves. All statistical comparisons were considered significant if *p* < 0.05.

## 3. Results

Genes encoding for proteins of the Wnt/PCP pathway, including VANGL1 and VANGL2, SCRIB, NOS1AP, receptor FZD7 and its ligands WNT5A and WNT11, PRICKLE and Dishevelled (DVL) homologues, Dishevelled Associated Activator of Morphogenesis 1 and 2 (DAAM1 and DAAM2) homologues, and ubiquitin ligase RNF41, show a low frequency of mutations in breast cancer. In the breast cancer cohort of TCGA with over a thousand patients, all PCP-related genes are mutated in less than 1% of cases (Table 1). None of these mutations have been evaluated for resulting protein functionality status; thus, their putative implications for oncogenesis are unknown. However, of 131 total mutations in PCP-related genes in the breast TCGA cohort, 25 mutations (19.1%) are nonsense mutations or frameshift mutations that are predicted to significantly affect the structure of the translated protein (Table 1).

**Figure 1:**
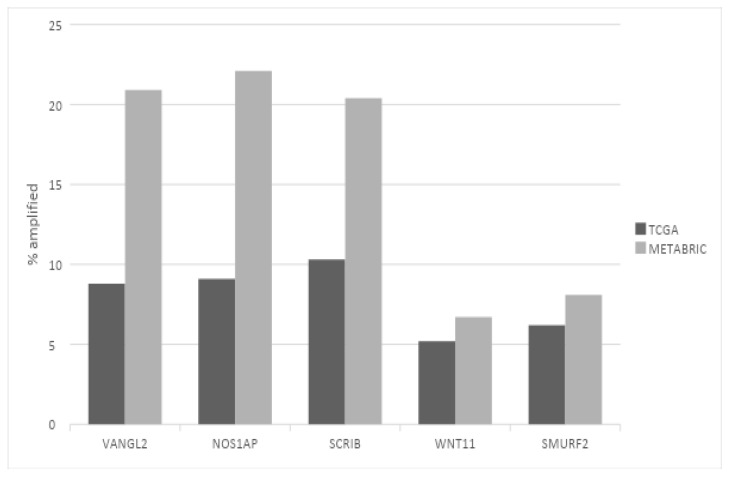
Prevalence of amplifications in the most frequently amplified planar cell polarity genes in breast cancer. Black bars: TCGA breast cancer cohort. Gray bars: METABRIC cohort. Fisher’s exact test *p* = 0.0001 for the VANGL2, NOS1AP and SCRIB comparisons; Fisher’s exact test *p* = 0.1 for the WNT11 comparison; Fisher’s exact test *p* = 0.04 for the SMURF2 comparison between the two datasets.

**Figure 2:**
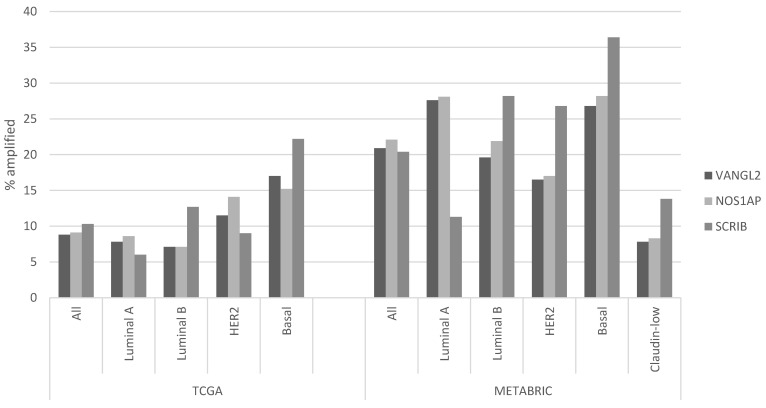
Prevalence of amplifications in *VANGL2*, *NOS1AP* and *SCRIB* according to breast cancer sub-type. The left part of the figure shows data from TCGA breast cancer cohort, and the right part of the figure shows data from the METABRIC cohort. Differences in the prevalence of all three gene amplifications in the breast cancer sub-types are statistically significant in both series (χ^2^ test *p* = 0.002 for the comparison of *VANGL2* in TCGA, χ^2^ test *p* = 0.02 for the comparison of *NOS1AP* in TCGA, and χ^2^ test *p* < 0.0001 for the comparison of *SCRIB* in TCGA and for all three genes in METABRIC series sub-types).

Regarding putative copy number alterations, while deep deletions are also rare, a few PCP-related genes are frequently amplified, as they are part of or are located close to prevalent breast cancer amplicons (Table 1). *VANGL2* and *NOS1AP* are located at neighboring loci 1q23.2 and 1q23.3 and are amplified in 8.8% and 9.1% of breast cancer cases of TCGA cohort, respectively (Figure 1). *SCRIB* is located at locus 8q24.3, close to oncogene *MYC*, and is amplified in 10.3% of TCGA breast cancer cases. In the METABRIC cohort, *VANGL2*, *NOS1AP* and *SCRIB* are amplified even more frequently, in 20.9%, 22.1% and 20.4% of cases, respectively (Figure 1). The subtype with the highest prevalence of amplifications of all three genes is the basal one (Figure 2). Over 20% of basal cancers in TCGA and 35% of basal cancers in METABRIC have amplifications of the *SCRIB* locus, consistently with the higher prevalence of *MYC* oncogene amplifications in these cancers. Co-amplifications of the VANGL2/NOS1AP and the SCRIB loci can be rarely observed (1.2% of cases), suggesting that those are separate events with different underlying pathophysiologic mechanisms and no additive benefit for bearing cells. Two other genes involved in PCP, ligand *WNT11* and ubiquitin ligase *SMURF2*, are amplified in smaller percentages of breast cancer cases (*WNT11*: in 5.2% of cases in TCGA and 6.7% of cases in METABRIC; *SMURF2*: in 6.2% of cases in TCGA and 8.1% of cases in METABRIC; Figure 1).

**Figure 3:**
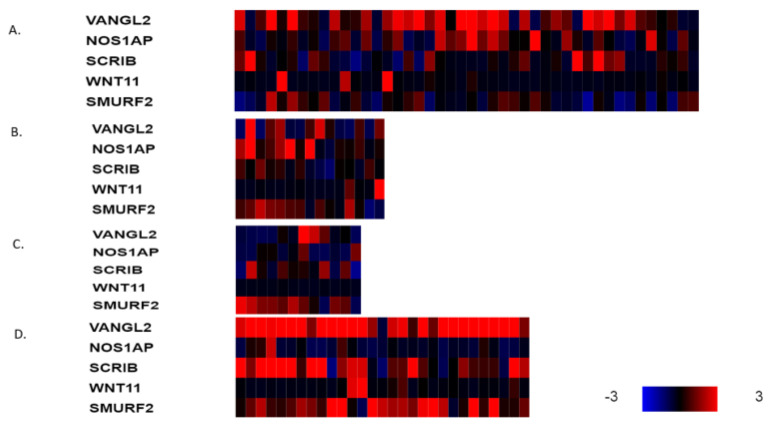
mRNA expression calculated as z-score relative to diploid samples (RNA Seq V2 RSEM) in breast cancer cases with *VANGL2* amplifications according to breast cancer sub-type: (**A**) luminal A cancers (*n* = 44), (**B**) luminal B cancers (*n* = 15), (**C**) HER2-positive cancers (*n* = 12) and (**D**) basal cancers (*n* = 29). Red denotes over-expression, and blue denotes suppression. Expression heat map key is presented in the lower right corner of the figure. Data are from TCGA.

**Figure 4:**
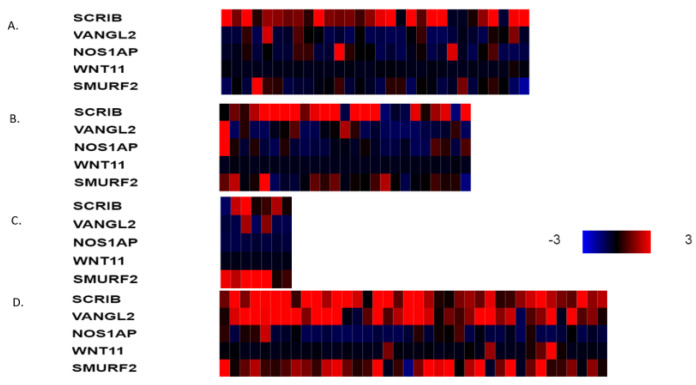
mRNA expression calculated as z-score relative to diploid samples (RNA Seq V2 RSEM) in breast cancer cases with *SCRIB* amplifications according to breast cancer sub-type: (**A**) luminal A cancers (*n* = 30), (**B**) luminal B cancers (*n* = 25), (**C**) HER2-positive cancers (*n* = 7) and (**D**) basal cancers (*n* = 38). Red denotes over-expression, and blue denotes suppression. Expression heat map key is presented on the right of the figure. Data are from TCGA.

**Figure 5:**
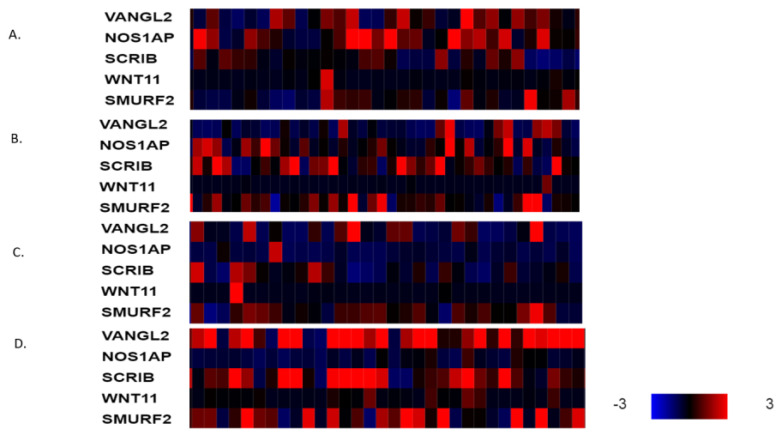
mRNA expression calculated as z-score relative to diploid samples (RNA Seq V2 RSEM) in representative breast cancer cases without *VANGL2* or *SCRIB* amplifications according to breast cancer sub-type: (**A**) luminal A cancers, (**B**) luminal B cancers, (**C**) HER2-positive cancers and (**D**) basal cancers. Red denotes over-expression, and blue denotes suppression. Expression heat map key is presented in the lower right corner of the figure. Data are from TCGA.

The mRNA expression of PCP-related genes *VANGL2*, *NOS1AP*, *SCRIB, WNT11* and *SMURF2* in *VANGL2*- or *NOS1AP*-amplified breast cancers (most cases are co-amplified) is heterogeneous in molecular sub-types. VANGL2 mRNA is over-expressed in many luminal A *VANGL2*/*NOS1AP*-amplified breast cancers and in most basal *VANGL2*- or *NOS1AP*-amplified breast cancers (Figure 3A,D). In contrast, luminal B and HER2-dependent *VANGL2*/*NOS1AP*-amplified breast cancers show over-expression of VANGL2 mRNA only occasionally (Figure 3B,C). NOS1AP mRNA is not consistently over-expressed in any of the four sub-types in *VANGL2*- or *NOS1AP*-amplified breast cancers (Figure 3A–D). Interestingly, SCRIBBLE and SMURF2 mRNAs are also over-expressed in several cases of basal *VANGL2*- or *NOS1AP*-amplified breast cancers but rarely in other subtypes. All sub-types of *SCRIB*-amplified breast cancers display mRNA over-expression of SCRIB (Figure 4A–D). *SCRIB*-amplified basal cancers show concomitant over-expression of VANGL2, which is not observed in other sub-types. Basal cancers without *VANGL2*, *NOS1AP* or *SCRIB* amplifications display frequent mRNA over-expression of VANGL2, SCRIB and SMURF2 (Figure 5D). Occasionally, these genes are over-expressed in the three other breast cancer sub-types (Figure 5A–C). These data imply that there is a lack of correlation between the copies of the genes present in the genome and corresponding mRNA transcript production. The regulation of planar polarity protein production and stability is complex in breast cancer cells and may include regulation at additional levels, such as transcription and protein turnover. Basal breast cancers frequently display over-expression of VANGL2 and SCRIB independently of the copy number status of their genes (Figure 3D, Figure 4D and Figure 5D).

**Figure 6:**
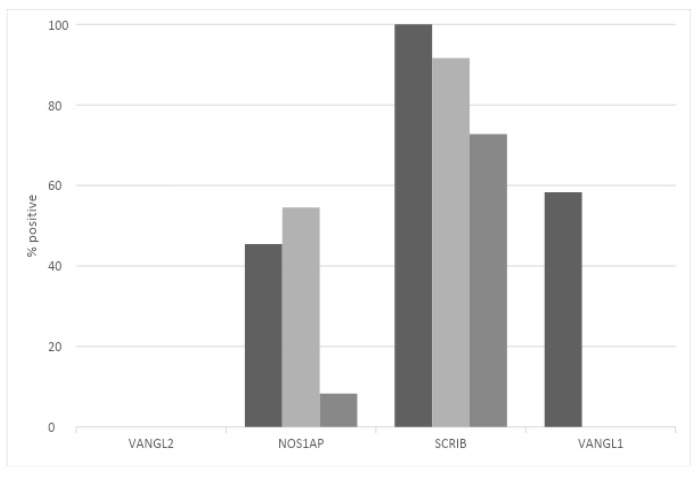
Prevalence of moderate-to-high protein expression of VANGL2, NOS1AP, SCRIBBLE and VANGL1 in breast cancer. Data are from the Human Protein Atlas.

At the protein level, SCRIBBLE is expressed with moderate intensity in the glands of normal mammary tissues, while the NOS1AP protein shows low expression, and the VANGL2 protein is not expressed, at least with the HPA027043 antibody used by the Human Protein Atlas. In contrast, its homologue VANGL1 shows positive expression, albeit at low levels. In breast cancers, the expression of SCRIBBLE is moderate in most cases with all three alternative antibodies (HPA023557, CAB015463 and CAB022081) used (Figure 6). The expression of NOS1AP is moderate in about half of the breast cancer cases with two of the three antibodies used (HPA030066 and HPA055561) and low to absent when a third antibody (CAB018582) was used. Of the two VANGL homologues, consistent with the results in normal mammary glands, VANGL1 is observed in half of the samples, while VANGL2 shows no staining (Figure 6). This analysis does not separate expressions in different breast cancer sub-types and thus cannot provide information regarding the correlation of mRNA expression, copy number alteration and protein expression of PCP proteins.

**Figure 7:**
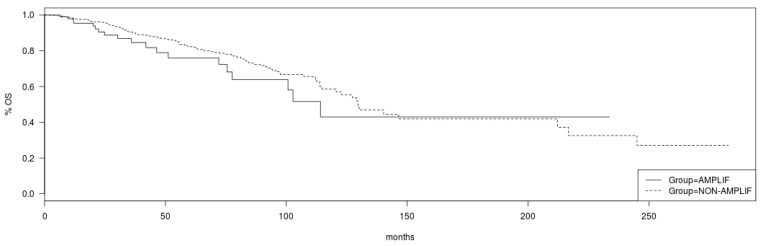
Overall survival of breast cancer patients with amplifications in the *VANGL2*/*NOS1AP* loci (solid line) versus non-amplified cases (interrupted line) (Log Rank test *p* = 0.19). Data are from TCGA cohort.

**Figure 8:**
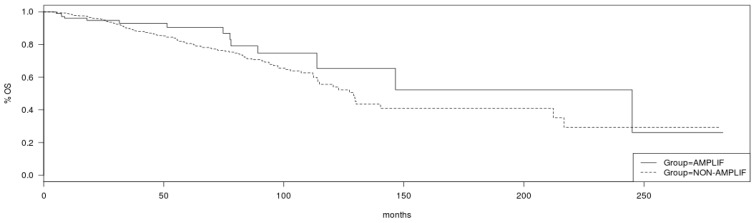
Overall survival of breast cancer patients with amplifications in the *SCRIB* locus (solid line) versus non-amplified cases (interrupted line) (Log Rank test *p* = 0.2). Data are from TCGA cohort.

Regarding survival implications of PCP alterations, although breast cancer patients in TCGA cohort with no amplifications in *VANGL2* or *NOS1AP* had a somewhat better OS than patients with amplifications in either or both genes, this difference does not reach statistical significance (Log Rank *p* = 0.19; Figure 7). Similarly, no statistically significant difference in OS of patients with and without amplification in *SCRIB* can be observed in TCGA cohort (Log Rank *p* = 0.2; Figure 8). mRNA expression of VANGL2, NOS1AP and SCRIB is not prognostic of survival in breast cancer as a whole or in the subsets of luminal A, luminal B, HER2 and basal cancers (Log Rank *p* corrected for multiple comparisons >0.05; not shown).

**Figure 9:**
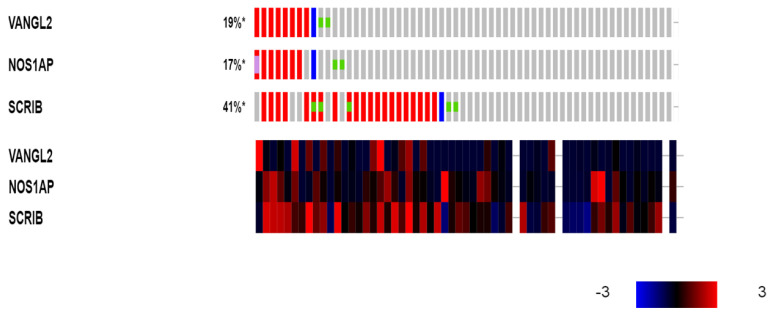
Genetic alterations (upper panel: red denotes amplifications; blue denotes deletions; and the green dots denote mutations) and mRNA expression (lower panel: red denotes over-expression, and blue denotes suppression) of VANGL2, NOS1AP and SCRIB in breast cancer cell lines. Expression heat map key is presented in the lower right corner of the figure. Data are from the Cancer Cell Line Encyclopedia. *: Percentages rounded to integers.

Several breast cancer cell lines in the CCLE collection display molecular defects in the three PCP genes amplified in clinical breast cancer samples. In total, 21 cell lines out of the 60 breast cancer cell lines of the CCLE (35%) possess amplifications in *SCRIB* (Table 2). Eight cell lines show amplifications in *VANGL2,* and seven of them show concomitant amplifications in the neighboring *NOS1AP* locus (Table 2, Figure 9). Four cell lines (CAMA1, BT-483, MDA-MB-483 and ZR-75-30) display amplifications in all three genes, *SCRIB*, *VANGL2* and *NOS1AP* (Table 3). In contrast to the rarity of mutations in clinical samples, a few breast cancer cell lines have mutations in one of the three genes, and two cell lines, including the commonly used MCF7 cell line, have deletions (Table 2). There is no significant correlation of amplifications in the three genes and the corresponding mRNA expression. Although most *SCRIB*-amplified cell lines show up-regulation of SCRIB at the mRNA level, several non-amplified cell lines also over-express mRNA levels of the gene, consistently with clinical samples (Figure 9).

The GDSC project includes two categories of drugs that inhibit the Wnt pathway. These are inhibitors of acyl-transferase porcupine, which performs the important step of acylation preceding the cellular secretion of Wnt ligands and the inhibitors of the enzymes tankyrase 1 and tankyrase 2 (also known as PARP5A and PARP5B), which promote the ubiquitination and degradation of axins, and are thus stabilizers of β-catenin. Breast cancer cell lines with *NOS1AP* locus amplifications (feature cnaBRCA64 in GDSC) show no differential sensitivity to either porcupine or tankyrase inhibitors compared with breast cancer cell lines without the amplification feature cnaBRCA64 (Table 4). Both cell lines with and without alterations in SCRIB, VANGL2 and NOS1AP are included among the most sensitive cell lines to the two classes of inhibitors.

Four breast cancer cell lines, CAMA1, BT-483, MDA-MB-436 and ZR-75-30, have amplifications in all three PCP genes of interest. These cell lines are of different breast cancer sub-types. CAMA1 and BT-483 are luminal; MBA-MD-436 is basal; and ZR-75-30 is ER positive and HER2 amplified (Table 3). CAMA1 is the only diploid cell line among the four. CAMA1 and MDA-MB-436 are sensitive to porcupine inhibitors assayed in GDSC but not to tankyrase inhibitors. Instead, CAMA1, which possesses a PTEN mutation, is sensitive to PI3K inhibitors, AKT kinase inhibitors and Estrogen Receptor inhibitors. CAMA1 also has a mutation in the CDH1 gene encoding for E Cadherin, a major regulator of β-catenin. Cell lines BT-483 and ZR-75-30 are drug pan-resistant, and despite being amplified for *SCRIB*, *VANGL2* and *NOS1AP*, they do not display sensitivity to porcupine or tankyrase inhibitors. The dependency evaluation of the cell lines with *SCRIB*, *VANGL2* and *NOS1AP* amplifications with CRISPR and RNAi revealed several genes involved in breast cancer pathogenesis, such as FOXA1, ERBB2, PIK3CA and CDK4, to be among the top preferential essential genes (Table 5). However, CRISPR and RNAi knockdown revealed no recurrent dependencies characterizing these cell lines with *SCRIB*, *VANGL2* and *NOS1AP* amplifications that could suggest therapeutic avenues in breast cancers with these amplifications.

## 4. Discussion

Cell polarity is a defining feature of epithelial cells, helping them to correctly interpret signals from neighboring cells [32]. The orientation of epithelial cells in monolayers across the basal–apical axis defines domains of the cell membrane that are closer to the surrounding matrix versus domains that are closer to the free cell surface. The perpendicular planar cell polarity (PCP) axis defines domains that are closer to the base versus the tip of crypts in glandular epithelia or the blind end versus the open end in ductal epithelia [16]. Orientation across the two axis receives cues from the environment through surface receptors ligated by ligands expressed in neighboring cells or by soluble ligands [33]. The role of planar cell polarity for cell movement extends from embryonic development to adult tissue homeostasis during physiologic turnover and post-injury healing, and its machinery is exploited by cancer cells for promotion of their mobility [34]. The asymmetry in the planar axis is established at the site of a homotypic interaction of the Flamingo-type atypical cadherins CELSR (Cadherin EGF LAG Seven-pass G-type Receptor) in two neighboring cells. In one cell, CELSR associates with a FZD receptor, and in the opposite cell, it associates with VANGL family tetraspanins. These two associations are mutually exclusive and establish the planar polarity and respective complexes consisting of FZD7/DVL/DAAM in one cell and VANGL/PRICKLE/RNF41 in the opposite, neighboring cell [16]. In addition, VANGL proteins serve as the docking site for SCRIB and NOS1AP, and the VANGL/SCRIB/NOS1AP complex promotes cell migration in breast cancer cells [17]. Interestingly, the VANGL/SCRIB/NOS1AP complex only assembles in cancer cells, while normal mammary epithelia show no co-localization of NOS1AP with either VANGL or SCRIB [17]. SCRIB is a component of the apical–basal cell polarity machinery; thus, it may serve as a communication hub for polarity and as a coordinator of motility on both planes in cancer cells [35]. The interaction of VANGL tetraspanins and SCRIB has been mapped to involve the carboxyterminal PBM (PDZ binding motif) domain of the former with the PDZ (PSD95-Discs large-ZO-1) domains of the latter [36].

Communication between the two opposing core complexes of PCP, FZD7/DVL/DAAM and VANGL/PRICKLE/RNF41, occurs at several nodes. Besides being a ligand for the FZD7 receptor after secretion, WNT5A protects VANGL from p97-promoted proteasome degradation in the endoplasmic reticulum, which is mediated by ubiquitin ligase KBTBD7 [37]. RNF41 and PRICKLE inhibit the association of DVL with the formin homology scaffold protein DAAM and their subsequent association with GTPase RhoA, which activates kinase ROCK to promote actin cytoskeleton and cell motility and migration [37]. DVL is also a component of canonical WNT/β-Catenin/APC pathway receptor complexes and thus provides a point of communication between canonical and non-canonical WNT/PCP transduction [38,39].

Given the accumulating evidence of the influence of planar polarity proteins in cancer and the metastatic process, the current investigation examined the expression of and alterations in an array of genes participating in PCP and their products in breast cancers. It is documented in the presented data that most PCP genes have no point mutations or copy number alterations in breast cancer. However, three PCP core genes, *VANGL2*, *NOS1AP* and *SCRIB*, are shown to have amplifications in 9–10% of cases in TCGA and 21–22% of cases in the different genomic platform used in the METABRIC cohort and have been selected for a more detailed evaluation [18,19]. Additional post-translational regulations lead to a poor correlation of amplifications and mRNA over-expression of the respective genes. Different sub-types show different patterns of mRNA expressions in these PCP players, with most basal cancers displaying up-regulation of SCRIB and VANGL2 mRNAs in both cases where they are amplified and non-amplified for the respective genes. In addition, SMURF2 mRNA, but not WNT11 mRNA, is often up-regulated in basal cancers. In contrast, luminal cancers show less consistent up-regulation of SCRIB and VANGL2 mRNAs and rare up-regulation of SMURF2 or WNT11. These data suggest a role of PCP alterations in the aggressive natural history of basal cancers, which may underline the greater metastatic potential of this sub-type [40,41]. The totality of these data supports the notion that a coordinated deregulation of SCRIB, VANGL2 and NOS1AP at the gene dosage or transcription level, possibly in conjunction with other PCP protein abnormalities, may affect cancer cell behavior in a sub-type-specific manner.

Despite these considerations, the survival analysis showed no differences in amplified or SCRIB, VANGL2 and NOS1AP mRNA-over-expressing breast cancers compared to non-amplified or not over-expressing counterparts. The lack of prognostic significance may result from the fact that key players in PCP may have a positive or negative influence on carcinogenesis processes. SMURF2, for example, is a negative regulator of PCP by promoting the degradation of PRICKLE proteins, while it is also a regulator of the TGFβ pathway, with roles in metastasis and epithelial–mesenchymal transition [42]. SCRIBBLE is both a positive effector in PCP and a major regulator of apical–basal polarity as part of a complex with DLG (Disc Large) and LLGL1 (Lethal Giant Larvae homologue), which has tumor suppressing functions [43]. In these roles, it represents a node of communication in polarity pathways on the two planes. It should also be noted that analyses of other series have disclosed worse survival outcomes in breast cancers over-expressing SCRIBBLE or VANGL1 mRNA [17].

Consistent with the results of the current study, SCRIBBLE has been shown to produce basal-type cancers when dislocated from cell–cell junctions in breast cancer cell models in which a mutant protein is expressed [44]. Cells with mutant SCRIBBLE exhibit the activation of the PI3K/AKT/mTOR pathway without possessing mutations in the genes encoding for the PI3K catalytic sub-unit or encoding for the phosphatase PTEN [44]. Despite the absence of *SCRIB* mutations in clinical breast cancer, over-expression resulting from amplifications in its gene or other mechanisms may increase the cytoplasmic localization of the protein and activate the PI3K/AKT/mTOR pathway. Moreover, amplifications in other members of the PCP complex may affect the stoichiometry of the interaction among them and lead to the delocalization of SCRIBBLE from the cytoplasm with the same effect on PI3K/AKT/mTOR pathway activation [45].

A study that examined SCRIBBLE protein expression using immunohistochemistry in histologic breast cancer samples confirmed the complex influence of SCRIBBLE on the prognosis of breast cancer, which depends on ER status and vimentin expression [46]. The influence of the stage of the disease on the prognostic implications of SCRIBBLE expression was also suggested, with patients expressing the protein in primary tumors but not in lymph node metastatic sites having a better prognosis than patients with SCRIBBLE expression in metastatic sites with or without expression in the primary tumor [46].

A limitation of the current study includes the fact that it describes data from previously performed experiments, and no additional “wet laboratory” experiments were performed for the purpose of clarifying putative dynamic interactions of the described PCP players. As a result, it is limited to a description of the status of the pathway at a single time point, when the analyzed material in the parent studies was obtained. This could be a significant hindrance in the understanding of a pathway that is, by its nature in the metastatic process, in constant flux. However, several of the described alterations in PCP, such as amplifications in a sub-set of its core genes, are not expected to be modified during the life cycle of the cancer cell. In addition, the examination of series with an extensive number of patients increases the confidence in the presented results.

The pharmacologic modulation of the Wnt pathway for the treatment of cancer has been studied, but no drugs targeting the pathway have been successful in entering the clinical arena to date [47]. Two classes of drugs, porcupine inhibitors and tankyrase inhibitors, affect processes regulating both β-catenin-dependent and β-catenin-independent WNT pathway transduction and thus can interfere with PCP [48,49]. Porcupine is the acyl-transferase that acylates WNT ligands, including ligands for the PCP receptors, a modification required for ligand secretion from the producing cell. In the absence of ligands in the extracellular space, FZD receptors are not engaged to start signal transduction [50]. Two of the four breast cancer cell lines with *SCRIB*, *VANGL2* and *NOS1AP* concomitant amplification in the CCLE collection display sensitivity to porcupine inhibitors, and one of them also shows sensitivity to drugs affecting the PI3K/AKT/mTOR pathway. Whether a combination of the two classes of drugs, porcupine inhibitors and PI3K pathway inhibitors, could have additional activity in breast cancers with defects in PCP proteins remains to be determined. It could also be interesting to investigate whether concomitant molecular aberrations in PI3K/AKT/mTOR pathway genes have an influence on the effectiveness of such combinations. In contrast to porcupine inhibitors, tankyrase inhibitors are not effective in the examined cell line models with PCP defects in vitro. This lack of effectiveness could be due to pharmacologic reasons but also to pharmacodynamic mechanisms related to other targets of tankyrases, besides the targets in WNT signaling, the axin proteins [51]. Tankyrase-mediated PARylation is involved in mitotic spindle formation and in DNA damage response [52,53]. More specific inhibitors that inhibit proteins and processes involved in PCP and devoid of roles in other pathways are preferable for the study of PCP inhibition in cancer and probably also for therapeutic exploitation. Inhibitors of FZD receptors could fulfill this requirement and have been developed. An anti-FZD antibody that inhibits FZD1, FZD2, FZD5, FZD7 and FZD8, vantictumab, was studied in combination with paclitaxel in a phase Ib trial in 48 patients with metastatic breast cancer [54]. Given its target receptors, vantictumab is an inhibitor of both β-catenin-dependent and -independent WNT pathways. A response rate of 31.3% and a clinical benefit rate of 68.8% were obtained, both considered encouraging in this population of patients, two thirds of whom were pretreated for their metastatic disease [54]. Despite the observed efficacy and overall acceptable tolerability of the combination, a worrisome incidence of fragility or traumatic fractures, occurring in three patients each, halted further development of vantictumab. A biomarker based on quantitative PCR measurement of six WNT pathway genes (CCND2, CTBP2, DKK1, FBXW2, RHOU and WIF1) was studied in an exploratory companion diagnostic development effort and was found to predict response to paclitaxel and vantictumab [54]. Similar biomarkers could help in the development of more specific inhibitors of the WNT/PCP pathway, which could also be devoid of skeletal toxicity. Drugs inhibiting the sub-set of FZD receptors serving primarily the non-canonical WNT/PCP pathway may be better tolerated, as the functions of the canonical WNT/β-catenin pathway are spared in normal tissues. The data presented in the current investigation suggest that the use of WNT/PCP pathway components as biomarkers of the efficacy of drugs targeting the pathway should be pursued, taking into consideration the breast cancer molecular sub-types.

## 5. Conclusions

The WNT/PCP pathway plays an important role in maintaining the identity of epithelial cells. Dysfunction of the pathway is observed in a sizable minority of breast cancers, stemming from copy number alterations and mRNA deregulation. This dysfunction represents a vulnerability of breast cancer cells and could form the basis for therapeutic manipulation in breast cancers.

## Figures and Tables

**Table 1:** Prevalence of alterations in planar cell polarity genes in breast cancer patients. Data are from TCGA. FSs: frameshift mutations.

Gene	Function	Mutations (%)	Missense	Nonsense	FSs	Amplifications (%)	Deletions (%)
WNT5A	Ligands of the β-catenin independent WNT pathway		0			0	4 (0.4)
WNT11		1 (0.1)	1	0	0	56 (5.2)	2 (0.2)
FZD7	Receptor of the β-catenin independent WNT pathway	2 (0.2)	1	1	0	2 (0.2)	5 (0.5)
VANGL1	Membrane tetraspanin	7 (0.7)	6	0	1	6 (0.6)	3 (0.3)
VANGL2	Membrane tetraspanin	8 (0.8)	8	0	0	94 (8.8)	0
PRICKLE1	Homologous scaffold proteins interacting with VANGL proteins and negative regulating DVL	7 (0.7)	3	2	2	10 (0.9)	0
PRICKLE2		6 (0.6)	4	2	0	0	6 (0.6)
PRICKLE3		5 (0.5)	0	0	0	10 (0.9)	1 (0.1)
PRICKLE4		4 (0.4)	3	0	0	18 (1.7)	3 (0.3)
DVL1	Scaffold proteins interacting with FZD receptors of the WNT/β-catenin-dependent and -independent pathways	3 (0.3)	3	0	0	3 (0.3)	10 (0.9)
DVL2		3 (0.3)	2	0	1	0	7 (0.7)
DVL3		3 (0.3)	3	0	0	22 (2.1)	0
DAAM1	Formin homology domain scaffolding proteins interacting with DVL and RhoA	10 (0.9)	8	0	1	8 (0.7)	0
DAAM2		12 (1.1)	9	0	1	11 (1)	2 (0.2)
SCRIB	Scaffold protein with PDZ domain	8 (0.8)	5	0	3	110 (10.3)	3 (0.3)
RNF41	Ubiquitin ligase, negative regulator of DVL	5 (0.5)	3	1	1	2 (0.2)	0
NOS1AP	Adaptor protein interacting with SCRIB involved in migration	5 (0.5)	5	0	0	97 (9.1)	0
RHOA	Small GTPase involved in actin cytoskeleton reorganization	8 (0.8)	6	0	2	1 (0.1)	5 (0.5)
ROCK1	Serine threonine kinase bound to GTP-bound RhoA	9 (0.8)	7	1	1	3 (0.3)	2 (0.2)
SQSTM1	Adaptor protein involved in endocytosis of VANGL proteins	5 (0.5)	3	0	1	10 (0.9)	2 (0.2)
MINK1	Kinase phosphorylating PRICKLE proteins promoting asymmetric localization	5 (0.5)	4	0	0	2 (0.2)	4 (0.4)
SMURF2	Ubiquitin ligase, negative regulator of PRICKLE	6 (0.6)	4	1	1	66 (6.2)	0

**Table 2:** Breast cancer cell lines with molecular lesions in *VANGL2*, *NOS1AP* and *SCRIB*. Data are from the Cancer Cell Line Encyclopedia.

Gene	Mutations	Amplifications	Deletions
SCRIB	HCC1395, HCC1569, KPL1, MCF7, MDA-MB-361	BT483, HCC1187, HCC1806 BT549, HCC1395, HCC1937, CAMA1, HCC1500, HCC2157, EFM19, HCC1569, HCC2218, HCC1143, HCC1599, HCC70, HDQ-P1, HS-578-T, MCF7, MDA-MB-436, UACC893, ZR7530	HCC1428
VANGL2	HCC1569, HS-343-T	BT483, CAL148, CAMA1, DU4475, HCC2157, HCC38, MDA-MB-436, ZR7530	MCF7
NOS1AP	HCC1143, MDA-MB-415	BT483, CAL148, CAMA1, DU4475, HCC38, MDA-MB-436, ZR7530	MCF7

**Table 3:** Characteristics of breast cancer cell lines with concomitant amplifications in VANGL2/NOS1AP and SCRIB loci.

Cell Line	DepMap ID	Type	Ploidy	Mutations/Mb
CAMA1	ACH-000783	ER+/HER2−, Luminal	1.93	47.03
BT-483	ACH-000818	ER+/HER2−, Luminal	3.84	46.45
MDA-MB-436	ACH-000573	ER−/HER2− Basal B	2.97	32.76
ZR-75-30	ACH-000828	ER +/HER2+ amplified	3.74	45.32

**Table 4:** Comparison of sensitivities of the groups of breast cancer cell lines with and without amplifications in 1q23.3 (feature cnaBRCA64) to porcupine and tankyrase inhibitors. The fourth column presents the number of cell lines with and without 1q23.3 amplifications. The fifth and sixth columns show the mean IC50 of the group of cell lines with 1q23.3 amplifications and without 1q23.3 amplifications, respectively. Some inhibitors were assayed only in GDSC2 dataset, while others were assayed in both GDSC1 and GDSC2 datasets.

Cell Line	Target	Dataset	N with/n without	Mean IC50 with (µM)	Mean IC50 without (µM)	*p*
IWP-2	PORCN	GDSC2	7/34	17.03	17.9	0.79
LGK974	PORCN	GDSC1	7/41	7.08	6.82	1
		GDSC2	9/39	44.97	52.21	0.69
Wnt-C59	PORCN	GDSC2	7/34	45.2	70.67	0.26
		GDSC1	7/41	6.78	6.63	0.88
AZ6102	Tankyrases 1 and 2	GDSC2	9/35	20.04	15.6	0.3
MN-64	Tankyrases 1 and 2	GDSC2	7/34	62.19	90.47	0.34
WIKI4	Tankyrases 1 and 2	GDSC2	9/35	68.49	38.54	0.36
XAV939	Tankyrases 1 and 2	GDSC2	7/34	106.47	85.35	0.24
		GDSC1	9/38	56.76	33.99	0.17

**Table 5:** Dependencies of breast cancer cell lines with VANGL2/NOS1AP and SCRIB amplifications. Genes with a prominent role in breast cancer pathogenicity are in bold.

Cell Line	CRISPR Preferential Essential Genes	RNAi
CAMA1	EIF1AX, YPEL5, TRPS1, UBE2H, PCYT1A, PSMB5, ARIH1, ACTB, NAMPT and GPX4	UBC, **MAP3K7**, **CDK4**, **FOXA1**, TFDP1, CIT, LRRC46, STRAP, MAGOHB and TRPS1
BT-483	NA	PREB, PTP4A1, NACAD, PITPNM1, HIGD1A, NEK11, POLM, NDC80, BTN3A1 and SNW1
MDA-MB-436	TYMS, INTS6, VPS4A, GPATCH1, DNAJC9, IER3IP1, WDR48, LEMD2, XRCC1 and ACO2	HSPA8, PPIL2, NSF, DHX8, CDC27, VPS4A, COPG1, TOPBP1, PCNA and WDR70
ZR-75-30	NA	MYL12B, UBA6, MED1, **PIK3CA**, CDC37, **FOXA1**, SRPRA, FSTL4, **ERBB2** and ASCC3

## Data Availability

There are no data available beyond data included in the manuscript.
